# Chlorophyll Breakdown in a Fern—Discovery of Phyllobilin Isomers with a Rearranged Carbon Skeleton

**DOI:** 10.1002/anie.201807818

**Published:** 2018-10-11

**Authors:** Theresia Erhart, Stefan Vergeiner, Christoph Kreutz, Bernhard Kräutler, Thomas Müller

**Affiliations:** ^1^ Institute of Organic Chemistry and Center for Molecular Biosciences University of Innsbruck Innrain 80–82 6020 Innsbruck Austria

**Keywords:** evolution, heterocyclic natural products, photosynthesis, porphyrinoids, rearrangements

## Abstract

All structure‐based information on chlorophyll (Chl) breakdown in the higher plants relies on studies with angiosperms. Herein, the first investigation of a fern is reported, revealing a novel type of Chl catabolites (phyllobilins) in leaves of this large division of the vascular plants, and providing structural insights into an astounding metabolic process of the higher plants that appears to have played a role even in early phases of plant evolution. The tetrapyrrolic Chl catabolites in the cosmopolitan bracken fern were discovered to be phyllobilin isomers with an unprecedented skeleton, proposed to be the striking result of a rearrangement of a hypothetical phyllobilin precursor.

Chlorophyll (Chl) breakdown is a major contributor to the emergence of the fall colors^1^ and a hallmark of leaf senescence and fruit ripening.^2^ The identification and unambiguous structure elucidation of a colorless tetrapyrrolic Chl catabolite from senescent leaves of barley was instrumental in promoting this field.^3^ Meanwhile, a variety of colorless Chl catabolites have been characterized as structurally related phyllobilins (PBs),^4^ and essential pieces of the puzzle of Chl breakdown have been revealed.2a, 5 However, so far, all structure‐based information on Chl breakdown in the higher plants relies on work with PBs from angiosperms,^2^, ^5^, ^6^ the contemporarily most diverse division of the plant kingdom.^7^ These studies have established the widely common PaO/phyllobilin pathway of Chl breakdown (Scheme 1),2a, 5b key “early” steps of which also occur in the green alga *Auxenochlorella protothecoides*, in a strikingly similar way.^8^ Hence, in spite of a lack of further structural support, Chl breakdown has been assumed to take place by the PaO/phyllobilin path in a wide range of land‐based photosynthetic organisms.^9^

**Scheme 1:**
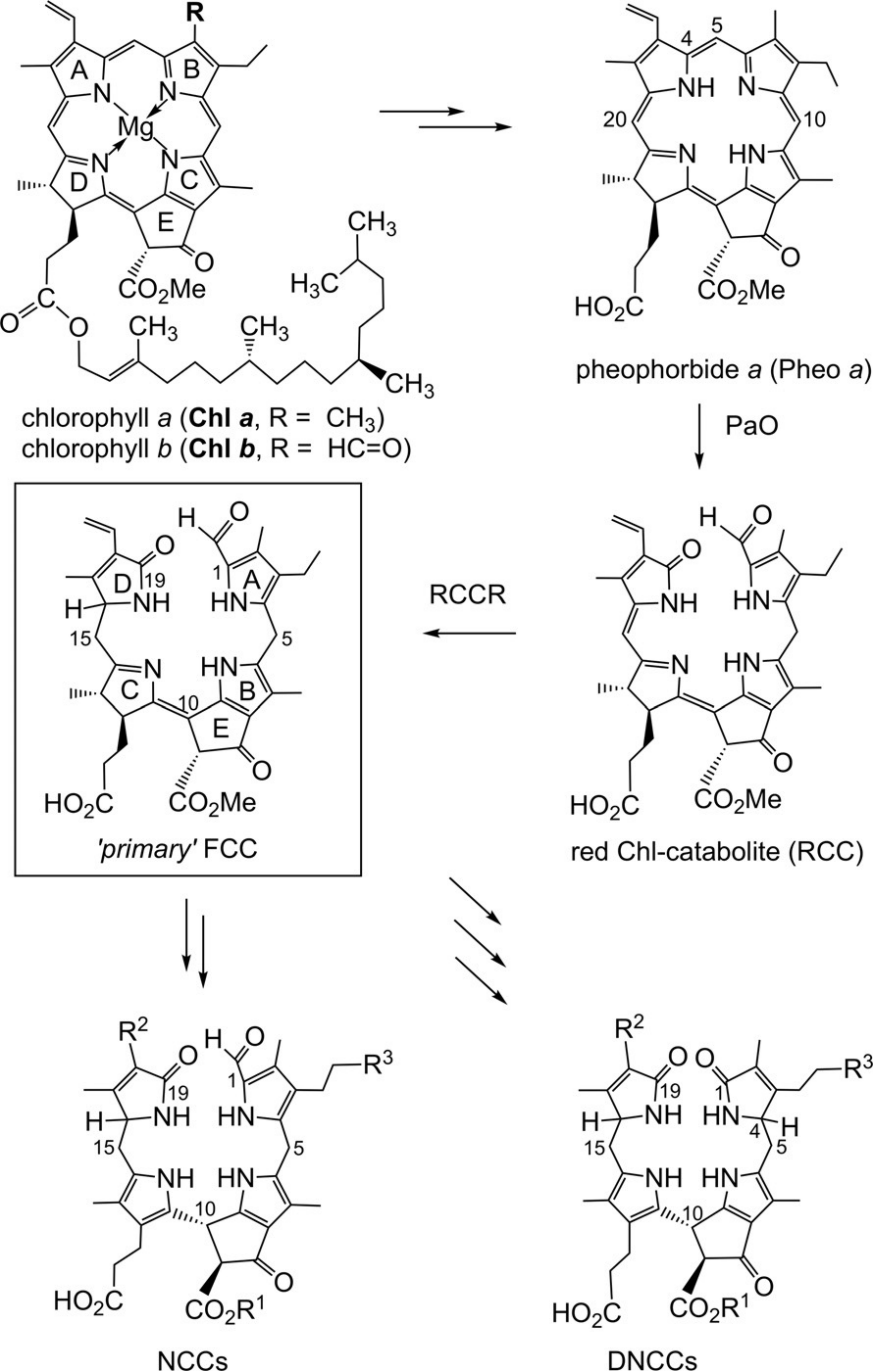
Structural outline of the PaO/phyllobilin pathway of Chl breakdown in angiosperms, in which the “primary” fluorescent Chl‐catabolite (“primary” FCC) is the first stage of a colorless phyllobilin. It is generated from the red Chl catabolite (RCC) by RCC reductase (RCCR). The “primary” FCC is converted further into colorless nonfluorescent Chl‐catabolites (NCCs) or to dioxobilin‐type NCCs (DNCCs), with various natural substituents R^1^, R^2^, and R^3^.5c The ring‐opening Pheo a oxygenase (PaO) and RCCR are highlighted as two key enzymes of the PaO/phyllobilin path.2a

Herein we report the first structures of tetrapyrrolic Chl catabolites in a fern that de‐greens and develops fall colors naturally (Figure 1). Strikingly, in fresh extracts of golden‐yellow fall leaves of the cosmopolitan bracken fern (*Pteridium aquilinum*), compounds with UV‐spectral properties of known Chl catabolites could not be detected by HPLC and LC/MS‐analyses. In particular no traces of the often abundant colorless phyllobilins were found, which have been classified as non‐fluorescent Chl catabolites (NCCs) or as dioxobilin‐type NCCs (DNCCs).5c

**Figure 1:**
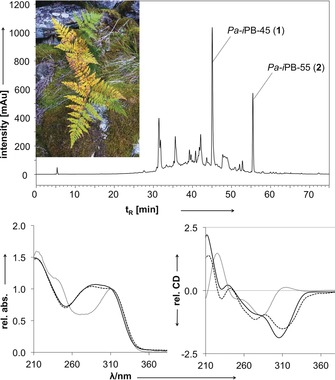
Top: HPLC‐analysis of an extract of senescent leaves of bracken fern (*Pteridium aquilinum*) leaves (detection at 320 nm), highlighting the identification of the two Chl catabolites *Pa‐i*PB‐45 (**1**) and *Pa‐i*PB‐55 (**2**). Inset: Picture of bracken with senescing fall leaves. Bottom: UV/Vis (left) and CD spectra (right) of Chl catabolites **1** (full line) and **2** (dashed line) from Bracken, and a colorless NCC^[5c, 10]^ (dotted line).

However, two conspicuous colorless fractions of the fern leaf extract displayed mass spectrometric features suggestive of tetrapyrrolic bilin‐type compounds, and a UV‐absorption near 315 nm, as is similarly found for authentic colorless NCCs^10^ (Figure 1). Remarkably, the UV/Vis spectra of both fractions showed additional bands near 280 nm.

We report herein on the structures of two Chl catabolites from bracken fern (*P. aquilinum*), provisionally named *Pa‐i*PB‐45 (**1**) and *Pa‐i*PB‐55 (**2**), as the first representatives of a novel phyllobilin category and classified herein as *iso*‐phyllobilanones (*i*PBs).

The unprecedented colorless *i*PB structures in the fern show the ring‐opened feature of the NCC analogues, the hallmark of the early steps of the PaO/phyllobilin pathway of Chl breakdown, but imply a distinctly different “later” path in this representative of the seedless higher plants,^7^ not revealed on the basis of the available bioinformatics tools.^9^

A crude extract obtained from 39.3 g (wet weight) of senescent leaves of common bracken was enriched in several steps and purified by semi‐preparative HPLC to furnish 3.9 mg of catabolite **1**, as well as 3.3 mg of catabolite **2**, as pale yellow powders (see Supporting Information for details).

In the ESI‐mass spectrum of the polar catabolite **1**, prominent ions at *m*/*z* 603.3 ([*M*+H]^+^) and *m*/*z* 625.3 ([*M*+Na]^+^) were observed (Figure S1 in the Supporting Information). The suggested molecular formula of C_33_H_38_N_4_O_7_ was confirmed by high‐resolution ESI mass spectrometry, showing a signal at *m*/*z* 603.2817 corresponding to the protonated molecular ion [C_33_H_39_N_4_O_7_]^+^ (*m*/*z*_calcd_ 603.2813). Collision‐induced fragmentation (CID) of the protonated molecular ion showed further surprising features (Figure S2). While loss of CO (−28 Da) was predominant, fragments from loss, either of MeOH (−32 Da) or of CO_2_ (−44 Da), as would be characteristic of the known natural colorless PBs, could not be detected.^11^

The 600 MHz ^1^H NMR spectrum of **1** in CD_3_OD (Figure S3) showed a set of characteristic signals of a bilin‐type tetrapyrrole moiety,^12^ with diagnostic structural elements inherited from the presumed precursor chlorophyll. Among them are four singlets at highfield assigned to four methyl groups (one of them, unexpectedly, shifted to 1.18 ppm), the coupled signals at 5.36, 6.14, and 6.49 ppm of a vinyl group, and a singlet at 9.51 ppm, arising from the presence of the characteristic formyl group. In addition, a new dd at the unusual chemical shift of 4.59 ppm, assigned to the C10 *meso*‐position, coupled with two multiplets near 2.74 and 3.17 ppm of a methylene group (assigned to C8^2^). The molecular constitution of *Pa‐i*PB‐45 (**1**) could be determined by multidimensional, homonuclear and heteronuclear NMR spectroscopy. ^1^H,^1^H‐ROESY‐ and ^1^H,^1^H‐COSY‐experiments, as well as ^1^H,^13^C‐HSQC‐ and ^1^H,^13^C‐HMBC‐spectra^13^ of a solution of **1** in CD_3_OH allowed the assignment of 34 H‐atoms and of all 33 C atoms and the unambiguous deduction of the constitution of **1** (Figure 2 and Scheme 2).

**Figure 2:**
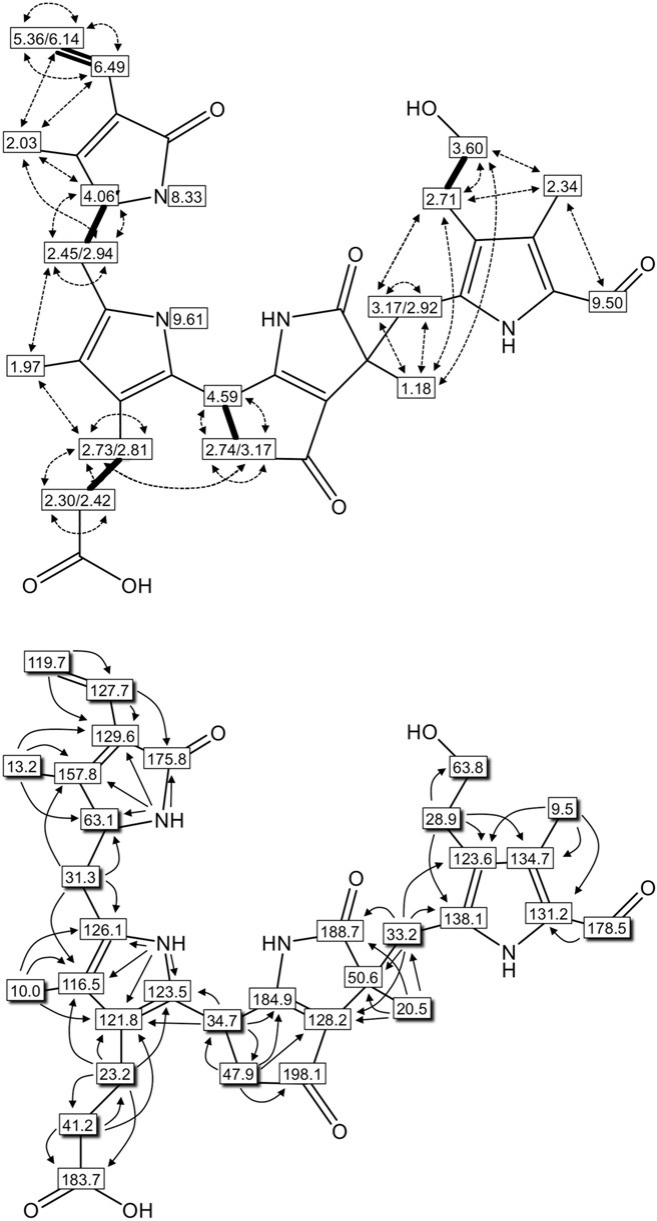
NMR chemical shift data of Chl catabolite *Pa‐i*PB‐45 (**1**) depicted in a graphical representation of the derived molecular structure of **1** (600 MHz NMR, CD_3_OH, 10 °C). Top: Correlations from ^1^H,^1^H‐ROESY experiment (dashed lines); bold bonds represent spin systems derived from the ^1^H,^1^H‐COSY spectrum. Bottom: Heteronuclear correlations based on assignments and obtained from ^1^H,^13^C‐HSQC spectra (shadowed boxes) and ^1^H,^13^C‐HMBC experiments (arrows).

**Scheme 2:**
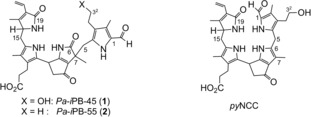
Formulae of *Pa‐i*PB‐45 (**1**), *Pa‐i*PB‐55 (**2**), and *py*NCC.

The ESI‐mass spectrum of the less‐polar catabolite **2** showed a prominent ion at *m*/*z* 587.3 (Figure S4). The suggested molecular formula of C_33_H_38_N_4_O_6_, with one oxygen atom less compared to the catabolite **1**, was again confirmed in a high‐resolution ESI mass spectrum, in which the protonated molecular ion [**2**+H]^+^ appears as a signal at *m*/*z* 587.2849, consistent with [C_33_H_39_O_6_N_4_]^+^ (*m*/*z*_calcd_ 587.2864). By collision‐induced fragmentation (CID) of the [**2**+H]^+^ ion, predominant loss of CO (−28 Da) was again observed, while neutral losses of MeOH or CO_2_ could not be detected (Figure S5). The 600 MHz ^1^H NMR spectrum of **2** in CD_3_OD (Figure S6) showed signals for 33 non‐exchangeable hydrogen atoms. In contrast to the spectrum of **1**, an A_3_X_2_ spin system was now present at 2.52 and 1.11 ppm in the spectrum of **2**, indicating the presence of an intact ethyl group assigned to the 3 position of the tetrapyrrolic core. The molecular constitution of **2** was unambiguously determined from a combination of ^1^H,^1^H‐ROESY‐, ^1^H,^1^H‐COSY‐, ^1^H,^13^C‐HSQC‐ and ^1^H,^13^C‐HMBC‐spectra, which allowed the assignment of all 33 non‐exchangeable H‐atoms and of all 33 C‐atoms (Figure S7). The constitution of **2** was thus deduced to match the one of the more polar catabolite *Pa‐i*PB‐45 (**1**), with the exception of the lack of the OH‐group of **1** attached to 3^2^ (Scheme 2).

To further secure the conclusions based on the NMR‐spectroscopic analysis of the Chl catabolites **1** and **2** from Bracken, *Pa‐i*PB‐45 (**1**) was treated with benzotriazol‐1‐yloxy‐tris(dimethylamino)‐phosphonium hexafluorophosphate (BOP) in methanol. The preparative outcome was the (formally) methylated derivative **3** of the Chl catabolite *Pa‐i*PB‐45 (**1**) with notably altered UV‐absorption properties and a new absorption maximum near 274 nm (Figure 3), owing to the formation of a new, conjugated iminoester function in **3**. Mass spectral analysis of **3** indicated a protonated molecular ion [**3**+H]^+^ at *m*/*z* 617.3, consistent with the deduced (formal) methylation. A fifth singlet at 3.67 ppm in the 600 MHz NMR spectrum of **3** and its heteronuclear correlations in ^1^H,^13^C‐HSQC and ^1^H,^13^C‐HMBC spectra revealed the new methyl group attached at an O‐atom of an iminoester function at ring B (Figure 3, Figures S11 and S12). These data indicated an O‐methylated iminoester function, derived from a novel unsaturated lactam function in a rearranged ring B part in the *iso*‐phyllobilanones **1** and **2**.

**Figure 3:**
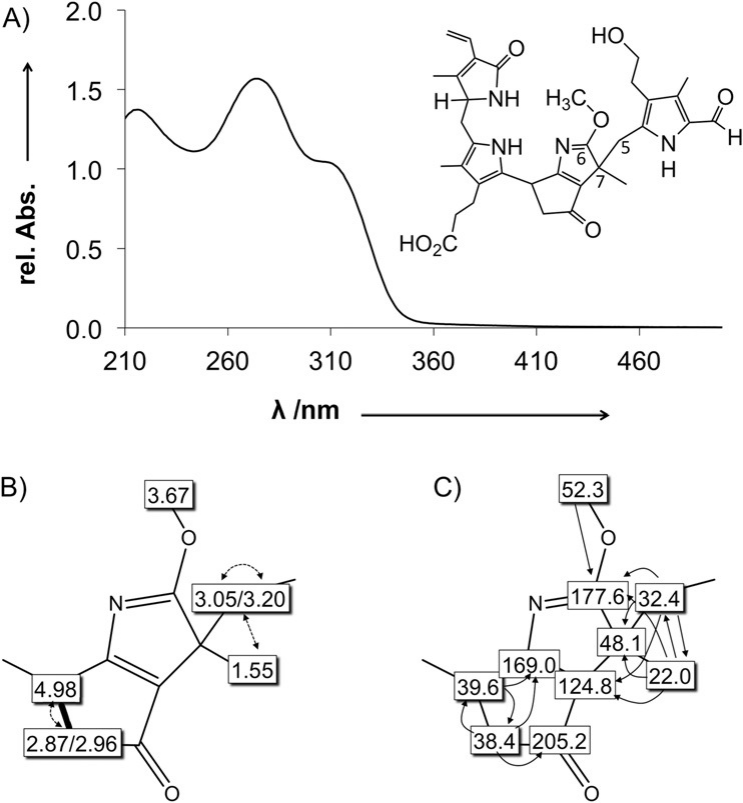
A) UV/Vis spectrum of iminoester **3** with formula of **3**. B) ^1^H,^1^H‐ROESY correlations (dashed lines) of the rings B and E; the bold bond represents the spin system derived from the ^1^H,^1^H‐COSY spectrum. C) Heteronuclear correlations based on assignments and obtained from ^1^H,^13^C‐HSQC spectra (shadowed boxes) and ^1^H,^13^C‐HMBC experiments (arrows) of the rings B and E.

The catabolites *Pa‐i*PB‐45 (**1**) and *Pa‐i*PB‐55 (**2**), as well as the iminoester **3**, were thus deduced to be related to NCCs, but to represent strikingly rearranged and oxidized phyllobilin isomers devoid of the typical carboxylic acid or ester function at C8^2^ of their ring E (Scheme 2). First representatives of phyllobilins that lack a carboxylic acid or ester function at C8^2^, so called pyro‐phyllobilins (*py*PBs), have been the subject of a recent study.^14^ In that work, a pyro‐NCC (*py*NCC) was prepared from a natural NCC with a ring E β‐ketocarboxylic acid group. The partial synthesis of the *py*NCC revealed the surprising resistance of the β‐ketocarboxylic acid group of the polar NCC against decarboxylation.^14^ Indeed, *py*NCCs are unknown as natural Chl catabolites and *py*PBs have only once been observed as the red products of an in vitro decarboxylation of natural red Chl catabolites excreted by the green alga *A. protothecoides*.^10^

The elucidation of the chemical constitution of the isophyllobilanones **1** and **2** raises intriguing questions concerning their natural formation as Chl catabolites. However, whereas many basic aspects of the path(s) of their formation remain obscure, key structural elements present in **1** and **2** are compatible with a role of an RCC8b, 15 and of FCCs^16^ as their precursors. Genomic analysis of ferns has, indeed, provided evidence for proteins related to the enzymes PaO^17^ and RCCR^18^ in these seedless higher plants,^9^ crucial for formation of hypothetical PBs, such as RCC and “primary” FCC. Hence, the biochemical pathway to FCC‐type intermediates may be available in the chloroplasts of ferns. In angiosperms, the PaO/phyllobilin pathway of Chl breakdown continues from “*primary”* FCCs to non‐fluorescent Chl catabolites (NCCs and DNCCs),^19^ and is presumed to pass through modified fluorescent FCCs, as the often hypothetical intermediates, generated from “*primary”* FCCs by enzymatic modifications.2a

The striking absence of a carboxylic acid or ester function in the isophyllobilanones **1** and **2** might indicate a role for a *py*RCC (or its carboxylic acid precursor8b) in Chl breakdown in the fern. Alternatively, the crucial oxidation step may occur correlated with the hypothetical decarboxylation, at the later stage of a colorless phyllobilin‐intermediate.

When considering the two major modifications separately, the α‐pyrrole position C6 of the known FCCs and NCCs, would also have the proper nucleophilic reactivity for the required enzymatic oxidation. Likewise, a β‐keto‐carboxylate, as present in some NCCs and FCCs, is activated (though only “modestly”^14^) for decarboxylation. Hence, enzyme catalyzed decarboxylation and pyrrole oxidation may presumably occur as separate steps during Chl breakdown in bracken ferns.

The hypothetical enzymatic hydroxylation at the pyrrolic α‐position C6 (or the formal equivalent of a transient epoxidation at C6–C7)^20^ of an FCC (or of an NCC) presumably triggers directly the striking carbon‐skeleton rearrangement, which is inferred for the formation of the isophyllobilanones, **1** and **2**. In this process, ring A and the C5‐methylene group would migrate as an integral, stabilized azafulvene fragment, and synchronized with the formation of the (protonated) vinylogous imide function of rings B and E (Scheme 3). The rearrangement of the hypothetical 6‐hydroxy‐PB is deduced to be strongly, thermodynamically driven by the formation of the resulting vinylogous imide function. Such a rearrangement has prominent precedence in the tetrapyrrole field, where the crucial biosynthetic formation of the type‐III porphyrinoids (from type‐I bilane precursors) proceeds by a spectacular enzyme‐catalyzed rearrangement at ring D of a hydroxybilane product, involving the (formal) migration of its ring C as an azafulvene unit.^21^ In a more general sense, the carbon‐skeleton rearrangement, proposed for the formation of the isophyllobilanones, **1** and **2**, may be considered an unprecedented variant of the rearrangement of a pinacol.^22^ This type of rearrangement has also served as mechanistic model for the unique enzymatic^21^, ^23^ and chemical^24^ ring contraction reactions generating corrins from the respective hydroporphyrinoid precursors.

**Scheme 3:**
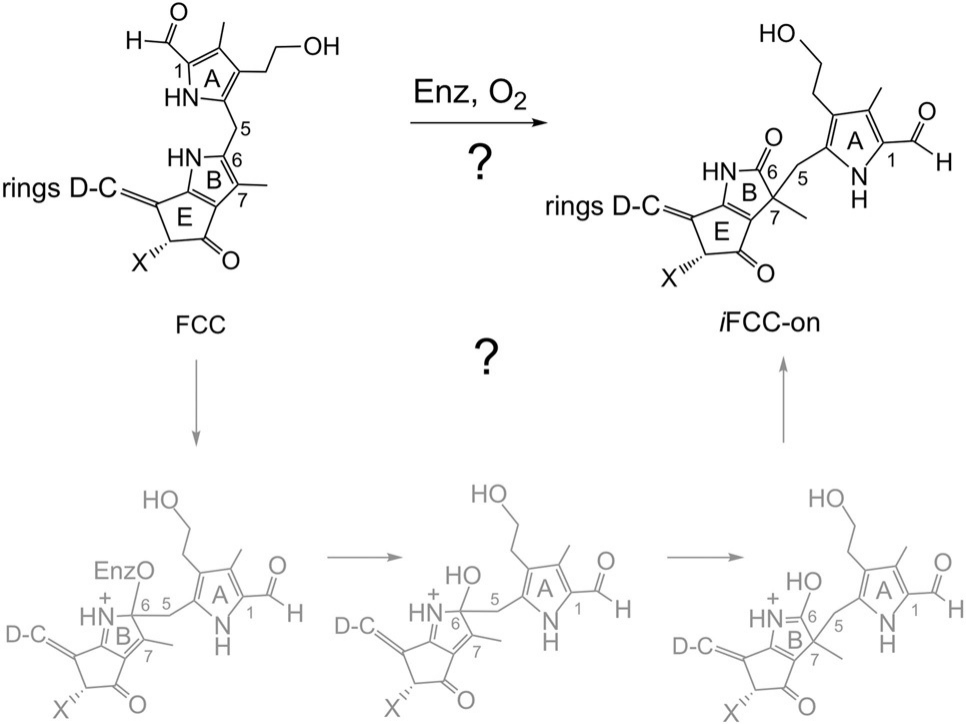
Hypothetical key rearrangement step in the oxidative transformation of PBs to *i*PBs. Oxidation at ring B of an FCC (X=CO_2_Me, CO_2_H or H) is proposed to take place in a correlated way with the migration of a ring A azafulvene moiety from C6 to C7 of ring B.

Further biochemical evidence concerning the PaO/phyllobilin path of Chl breakdown, as well as the presence of both of the isophyllobilanones, **1** and **2**, suggest, both, the hypothetical oxidation at C6 to be a process competing with hydroxylation of a *“primary”* FCC at C3^2^ (in *A. thaliana*, this hydroxylation is catalyzed by the plastidic enzyme TIC55^24^). Hence, the hypothetical PB‐oxidation at C6 would probably occur at the level of an FCC, and with molecular oxygen as the presumed co‐substrate (Scheme 3). This is the stage at which the oxygen‐dependent enzymatic peripheral oxidation by the hydroxylase TIC55^25^ takes place in the PaO/phyllobilin path of Chl breakdown.2a, 5b

The discovery of Chl catabolites in senescent leaves of bracken fern has allowed a first glimpse into Chl breakdown in a seedless plant that is considered evolutionarily less advanced than the flowering plants,^7^ which, so far, represent the only division of the plant kingdom to be investigated with respect to the intriguing biochemical path of Chl breakdown.2a, 5 The structures revealed of the isophyllobilanones **1** and **2** reflect the presumed operation of the early steps of the PaO/phyllobilin pathway of Chl breakdown in the fern, supported by bioinformatics data,^9^ but imply a distinctly different subsequent path, involving an unprecedented extra oxidation process. For this step molecular oxygen is presumably required as a co‐substrate that is generated in the thylakoids by photosynthesis.^26^ As Chl is phototoxic in the presence of O_2_,2a efficient Chl degradation occurs during leaf senescence.^25^ Breakdown of Chl is also the source of a variety of phyllobilins, abundant heterocyclic natural products that may have beneficial physiological effects to the plants.2a, 27 Hence, the intricate diversity of the pathways of Chl degradation may reflect the vast potential of plants to produce tetrapyrrolic compounds of hypothetical physiological relevance as particular complex heterocycles.5c

Our work has allowed first structure‐based insights into the fate of Chl in senescent leaves of a fern, helping to close an intriguing knowledge gap concerning Chl breakdown,5b a basic metabolic process in vascular plants that appears to have played a role in Chl photobiology in geologically old phases of the evolution of plants.^9^ Strikingly, for the so far elusive Chl catabolites in senescent tissues of the unique maiden hair tree, *Ginkgo biloba*, and of some conifers, such as European larch (*Larix decidua*), spectroanalytical evidence supports a rearranged *iso*‐phyllobilin carbon skeleton, related to the one described herein. The structures of all of these novel Chl catabolites, which are the subject of ongoing further investigations, will expand the available information concerning Chl breakdown in the plant kingdom.

## Experimental Section

See the Supporting Information for details concerning the collected plant material, HPLC analyses, isolation of *Pa*‐*i*PB‐45 (**1**) and *Pa*‐*i*PB‐55 (**2**), transformation of **1** into iminoester **3**, as well as spectroscopy.

*Spectroscopy*: **1**: UV/Vis (MeOH, *λ*_max_ nm^−1^ (rel *ϵ*)): 213 (1.49), 290 (1.07), 311 nm (sh, 1.00); CD (MeOH): *λ*_max_ nm^−1^ (Δ*ϵ*)=212 nm (1.2); 230 nm (0.1); 287 nm sh (−0.55), 310 nm (−1.0) (Figure 1). ESI‐HRMS (Bruker FT‐ICR): *m*/*z* 603.2817 ([**1**+H]^+^; *m/z*_calcd_[C_33_H_39_N_4_O_7_]^+^ 603.2813). **2**: UV/Vis (MeOH): *λ*_max_ nm^−1^ (rel *ϵ*))=213 (1.48), 286 (1.04), 311 nm (sh, 1.00); CD (MeOH): *λ*_max_ nm^−1^ (Δ*ϵ*)=212 nm (0.93); 230 nm (0.08); 287 nm sh (−0.70), 310 nm (−1.0) (Figure 1). ESI‐HRMS: *m*/*z* 587.2849 ([**2**+H]^+^; *m/z*_calcd_[C_33_H_39_N_4_O_7_]^+^ 587.2864). **3**: UV/Vis (MeOH): *λ*_max_ nm^−1^ (rel *ϵ*))=216 (1.37), 274 (1.57), 314 nm (sh, 1.00); ESI‐HRMS: *m*/*z=*617.2925 ([**3**+H]^+^; *m/z*_calcd_[C_34_H_41_N_4_O_7_]^+^ 617.2970).

## Conflict of interest

The authors declare no conflict of interest.

## Supporting information

As a service to our authors and readers, this journal provides supporting information supplied by the authors. Such materials are peer reviewed and may be re‐organized for online delivery, but are not copy‐edited or typeset. Technical support issues arising from supporting information (other than missing files) should be addressed to the authors.

Supplementary
