# Supplementary material for: Chlorophyll Breakdown in a Fern—Discovery of Phyllobilin Isomers with a Rearranged Carbon Skeleton
Source: Angew Chem Int Ed Engl. 2018 Oct 11;57(45):14937–41. doi: 10.1002/anie.201807818 (PMC6220952; doi:10.1002/anie.201807818)
Supplement: Supplementary file 1 — Supplementary [file ANIE-57-14937-s001.pdf]

Angewandte Chemie
Eine Zeitschrift der Gesellschaft Deutscher Chemiker

Supporting Information

Chlorophyll Breakdown in a Fern—Discovery of Phyllobilin Isomers with a Rearranged Carbon Skeleton

Theresia Erhart[+], Stefan Vergeiner[+], Christoph Kreutz, Bernhard Kräutler, and Thomas Müller**

anie_201807818_sm_miscellaneous_information.pdf

Author Contributions

T.E. Investigation: Equal; Visualization: Equal
S.V. Investigation: Equal; Visualization: Equal
C.K. Data curation: Supporting; NMR: Supporting
B.K. Writing – original draft: Equal; Writing – review & editing: Equal
T.M. Conceptualization: Lead; Investigation: Lead; Methodology: Lead; Project administration: Lead; Writing – original draft: Equal; Writing – review & editing: Equal.

Materials.

Leaves of bracken fern (*Pteridium aquilium; Pa*) were collected at different stages of senescence near the Hungerburg district of Innsbruck, Austria, and were stored at -20 °C before further usage.
Commercial solvents were either distilled (reagent grade) or directly used (HPLC grade) for extractions. HPLC-grade MeOH was purchased from VWR Chemicals, n-Hexane Dichloromethane (technical grade), Triethylamine, Potassium dihydrogen phosphate, *puriss. p.a.*, Potassium phosphate dibasic-anhydrous, *puriss. p.a*. from Sigma-Aldrich. Ammonium acetate, *puriss. p.a.,* was from Fluka, and Benzotriazol-1-yloxy-tris(dimethylamino)-phosphonium hexafluorophosphate (BOP) from Bachem. Ultrapure water (18 $M\Omega*cm^{-1}$) was from Millipore apparatus. SepPak cartridges (5g) were from Waters Associates.

Methods.

Analytical HPLC: Shimadzu HPLC system equipped with an LC-20AD pump, a DGU-20A5 online degasser unit and an SPD-M20A diode array detector, a Rheodyne injection valve with a 20 µl loop; column: Phenomenex Hyperclone column (ODS 5 µm 250x4.6 mm i.d., connected to a Phenomenex ODS 4x3 mm i.d. precolumn); flow rate was 0.5 $mL*min^{-1}$; solvent A: 50 mM potassium phosphate buffer (pH 7), solvent B: MeOH; solvent composition A/B (v/v): 0-5 min: 80/20; 5-55 min: 80/20 to 30/70; 55-60 min: 30/70 to 0/100; 60-70 min: 0/100; 70-75 min: 0/100 to 80/20). Data were collected and processed using Shimadzu LC Solution software (version 1.24 SP1).

Semipreparative HPLC for the isolation of *Pa-i*PB-45 and *Pa-i*PB-55: Shimadzu HPLC system equipped with an LC-20AD pump, a DGU-20A5 online degasser unit and an SPD-M20A diode array detector, a Rheodyne injection valve with a 6 mL loop; column: Phenomenex Hyperclone column (ODS 5 µm 250x21.2 mm i.d., connected to a Phenomenex ODS 4x3 mm i.d. precolumn); flow rate was 5 $mL*min^{-1}$; solvent A: 50 mM potassium phosphate buffer (pH 7), solvent B: MeOH; solvent composition A/B (v/v): 0-5 min: 80/20; 5-10 min: 80/20 to 55/45; 10-130 min: 55/45 to 45/55; 130-135 min: 45/55 to 0/100; 135-150 min: 0/100; 150-155 min: 0/100 to 80/20. Data were collected and processed using Shimadzu LC Solution software (version 1.24 SP1). For the isolation of *Pa-i*PB-45 iminoester: Dionex UltiMate 3000 HPLC system equipped with an UltiMate 3000 pump, an online degasser, a diode array detector and a Rheodyne injection valve with a 200 µl loop; column: Phenomenex Hyperclone ODS 5 µm 250x4.6 mm i.d. column with a Phenomenex ODS 4x3 mm i.d. pre-column; flow rate: 0.5 $mL*min^{-1}$; solvent A: 50 mM potassium phosphate buffer (pH 7), solvent B: methanol; solvent composition A/B (v/v): 0-5 min: 45/55; 5-20 min: 45/55 to 42/58; 20-23 min: 42/58 to 0/100; 23-23 min: 0/100; 26-30 min: 0/100 to 45/55. Data were collected and processed using Chromeleon 6.50.

Ultraviolet/visible (UV/Vis) spectroscopy: Agilent Technologies spectrophotometer (type: Cary 60 UV-Vis), in MeOH; λ_{max} [nm] (ε_{rel}); 10 x 10 mm UV-cells (quartz).

Circular dichroism (CD) spectroscopy: JASCO (type: J715), in MeOH, $\lambda_{min/max}$ [nm] (ε_{rel}); 10 x 10 mm UV-cells (quartz).

^{1}H- and ^{13}C-Nuclear Magnetic Spectroscopy (NMR): Bruker (type: Ultrashield 600 MHz) or Varian (type: UNITYplus 500 MHz) spectrometer, δ in ppm with δ (C^1HD_2OD) = 3.31 ppm and δ ($^{13}CD_3OD$) = 49.0 ppm[1], J_{HH} (Hz); ^{13}C- signal assignment from heteronuclear ^{1}H- and ^{13}C-HSQC & HMBC experiments.
High resolution (HR) mass spectrometry (MS): Bruker Apex Ultra Fourier-Transformation-Ion-Cyclotron-Resonance (FT-ICR) spectrometer at 7 Tesla, equipped with an Electrospray Ionization (ESI) source (positive-ion mode, spray voltage 4 kV, solvent: water/MeOH 1:1 (v/v), polypropylene glycol as internal mass standard). Data were collected and processed with Bruker Data analysis software. ThermoScientific LTQ Orbitrap XL mass spectrometer, equipped with an ESI source (positive-ion mode, spray voltage 4.5 kV, solvent: MeOH/4 mM ammonium acetate. Data were collected and processed with Xcalibur 2.2 software.

Analytical HPLC analysis of senescent bracken fern.

2 g (wet weight) of greenish-yellow bracken leaves were deep-frozen in liquid nitrogen and ground in a mortar together with 0.2 g of sea sand and 4 mL of methanol (MeOH). After centrifugation, the yellow supernatant was diluted with water 1:5 (v/v) and loaded onto a 1g SepPak cartridge. After washing with 30 mL of 20% MeOH a light orange fraction was eluted with 5 mL of MeOH. An aliquot of 20 µL of the eluate was subjected to analytical reversed-phase (RP) HPLC (see Figure 1 in the main text).

Isolation of *Pa-i*PB-45 (1) and *Pa-i*PB-55 (2).

39.3 g of senescent, yellowish-green Bracken leaves were deep-frozen in liquid nitrogen and ground in a mortar together with 2 g sea sand. The resulting powder was extracted using 300 mL of a cold mixture (4 °C) of dichloromethane/methanol (9:1; v/v). Under occasional stirring the suspension was then allowed to warm up to r.t. The slurry was filtered through a Buchner funnel and the filter cake was washed with 100 mL of a mixture of dichloromethane/methanol (9:1; v/v). After the addition of 50 g silica 60 to the collected filtrate, the obtained suspension was filtered through a Buchner funnel and the silica residue was washed with 100 mL of a mixture of dichloromethane/methanol (9:1; v/v). To obtain a fraction containing a concentrated crude extract, the remaining filter cake was washed with 250 mL of methanol. After the methanol had been evaporated under vacuo, the residue was re-dissolved in 20 mL of methanol, diluted with 40 mL potassium phosphate buffer (50 mM, pH 6.9) and centrifuged at 4000 x g for 10 min. 30 mL aliquots of the clear supernatant were subjected to desalting using a SepPak-cartridge (5g). After washing with 20 mL H_2O a fraction containing the crude product was eluted with using 10 mL methanol. The methanolic fractions were combined and the solvents evaporated.
The residue was re-dissolved in 8 mL of a mixture of methanol/potassium phosphate buffer 50 mM at pH 7 (1:4; v/v) and centrifuged at 5000 rpm for 10 min. The clear supernatant was subjected to semi-preparative HPLC. A fraction containing *Pa-i*PB-45 were collected from R_t = 47 to 52 min, the fraction containing *Pa-i*PB-55 were collected from R_t = 107 to 116 min. After desalting using a SepPak-C18 cartridge (5g) and removal of the solvents in vacuo, 3.9 mg pure *Pa-i*PB-45 as well as 3.3 mg pure *Pa-i*PB-55 were obtained for further spectroscopic analyses. All operations were performed under light-protection.

Spectroanalytical data of *Pa-i*PB-45 (1).

HPLC: Retention time in analytical HPLC (t_R)=45 min

ESI-MS *m/z* (%): 663.5 (7, $[M-H+K+Na]^+$); 647 (7, $[M-H+2Na]^+$); 641.2 (27, $[M+K]^+$); 627.3 (4), 626.3 (36), 625.3 (100, $[M+Na]^+$); 605.3 (2), 604.3 (21), 603.3 (61, $C_{33}H_{39}N_4O_7^+$, $[M+H]^+$); 575.3 (18, $[M-CO+H]^+$); 480.2 (42, $[M-C_7H_9NO+H]^+$); 452.2 (25, $[M-CO-C_7H_9NO+H]^+$); 315.1 (9).

ESI-HRMS (Bruker FT-ICR): m/z=587.2849 ($[\mathbf{2}+H]^+$; $m/z_{calc}[C_{33}H_{39}N_4O_7]^+$= 587.2864).

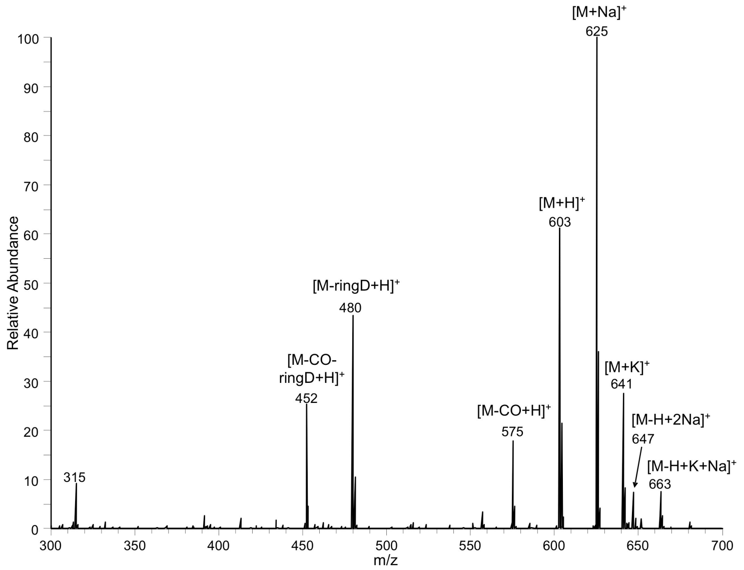

Figure S1. ESI-mass spectrum of ***Pa-i*PB-45 (1)**.

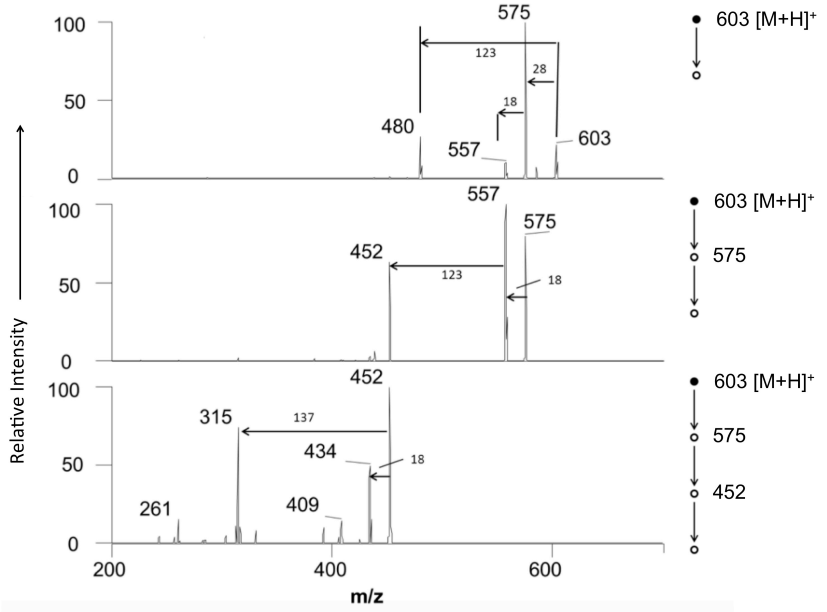

Figure S2. Fragmentation the isolated $[M+H]^+$ ion of ***Pa-i*PB-45 (1)**.

^{1}H-NMR (600 MHz; in CD_3OH) at 10 °C, signal assignment from ^{1}H,^{13}C-HSQC, ^{1}H,^{13}C-HMBC, ^{1}H,^{1}H-COSY and ^{1}H,^{1}H-ROESY: δ [ppm] = 1.18 (s, H_3C-7^1), 1.97 (s, H_3C-13^1), 2.03 (s, H_3C-17^1), 2.30 (m, H_AC-12^2), 2.34 (s, H_3C-2^1), 2.45 (m, H_AC-15 superimposed by 2.42 (m, H_BC-12^2 diastereotopic), 2.71 (m, H_2C-3^1 superimposed by 2.73 (m, H_AC-12^1) and 2.74 (m, H_AC-8^2)), 2.81 (m, H_BC-12^1), 2.92 (m, H_AC-5 superimposed by 2.94 (m, H_BC-15)), 3.17 (m, H_BC-5 superimposed by 3.19 (m, H_BC-8^2)), 3.60 (m, H_2C-3^2), 4.06 (dd, *J*=4.3/9.6 Hz, HC-16), 4.59 (dd, *J*=1.8/6.8 Hz, HC-10), 5.36 (dd, *J*=2.2/11.7, H_AC-18^2), 6.14 (dd, *J*=2.2/17.8 Hz, H_BC-18^2), 6.49 (dd, *J*=11.7/17.8, HC-18^1), 8.33 (s, HN-24), 9.50 (s, HC-20), 9.61 (s, HN-23).

^{13}C-NMR (150 MHz; in CD_3OH) at 10 °C, signal assignment from ^{1}H,^{13}C-HSQC and ^{1}H,^{13}C-HMBC: δ [ppm] = 9.5 (2^1), 10.0 (13^1), 13.2 (17^1), 20.5 (7^1), 23.2 (12^1), 28.9 (3^1), 31.3 (15), 33.2 (5), 34.7 (10), 41.2 (12^2), 47.9 (8^2), 50.6 (7), 63.1 (16), 63.8 (3^2), 116.5 (13), 119.7 (18^2), 121.8 (12), 123.5 (11), 123.6 (3), 126.1 (14), 127.7 (18^1), 128.2 (8), 129.6 (18), 131.2 (1), 134.7 (2), 138.1 (4), 157.8 (17), 175.8 (19), 178.5 (20), 183.7 (12^3), 184.9 (9), 188.7 (6), 198.1 (8^1).

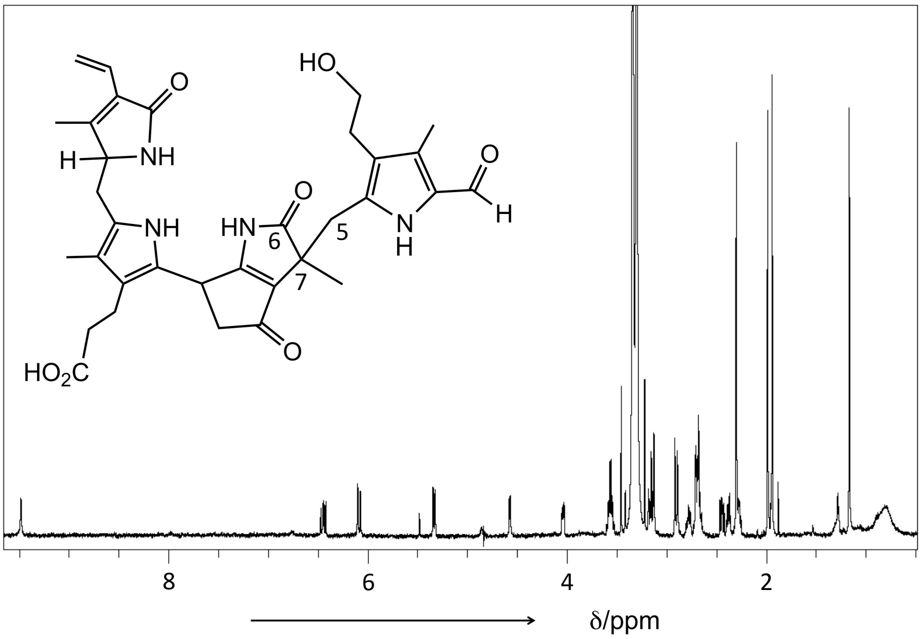

Figure S3. 600 MHz ^{1}H NMR spectrum of *Pa-i*PB-45 (1) (CD_3OD, 10 °C) and constitutional formula of **1** derived from detailed homonuclear and heteronuclear NMR-spectral analysis.

Spectroanalytical data of *Pa-i*PB-55 (2).

HPLC: Retention time in analytical HPLC (t_R)=55 min

ESI-MS *m/z* (%): 663.5 (12, $[M\text{-}H\text{+}2K]^+$); 625.2 (40, $[M\text{+}K]^+$); 609.3 (54, $[M\text{+}Na]^+$); 589.3 (3), 588.3 (34), 587.3 (100, $C_{33}H_{39}N_4O_6^+$, $[M\text{+}H]^+$); 559.3 (34, $[M\text{-}CO\text{+}H]^+$); 464.2 (68, $[M\text{-}C_7H_9NO\text{+}H]^+$); 445.2 (5); 436.2 (42, $[M\text{-}CO\text{-}C_7H_9NO\text{+}H]^+$); 315.1 (14).

ESI-HRMS (ThermoScientific LTQ Orbitrap XL): *m/z*=617.2925 ($[3\text{+}H]^+$; $m/z_{calc}[C_{34}H_{41}N_4O_7]^+$= 617.2970).

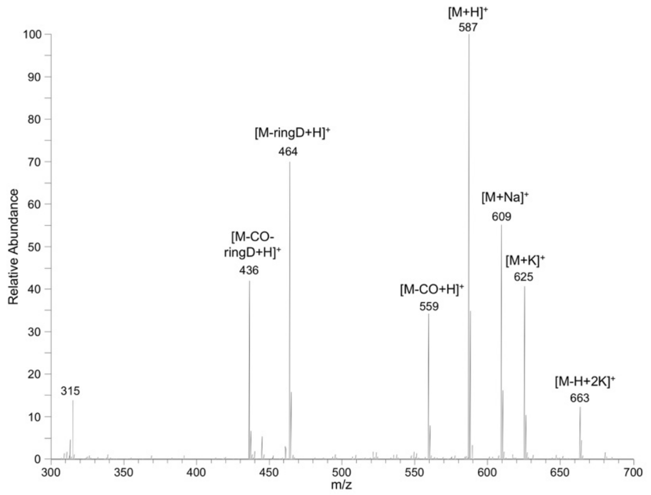

Figure S4. ESI-mass spectrum of the catabolite ***Pa-i*PB-55 (2)**.

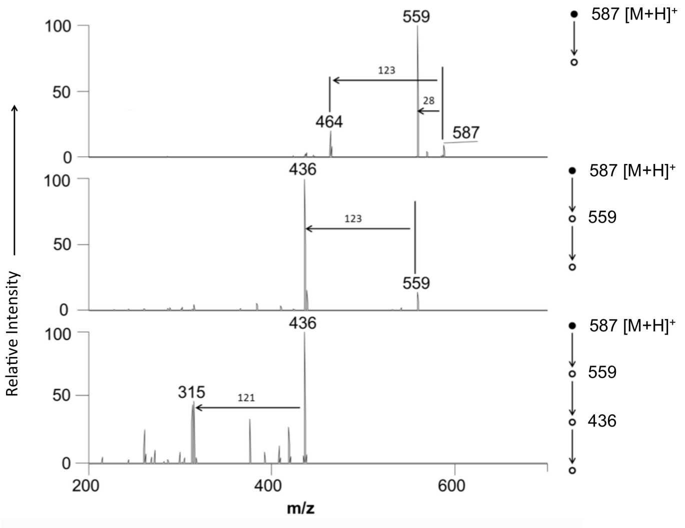

Figure S5. Fragmentation the isolated $[M\text{+}H]^+$ ion of the catabolite ***Pa-i*PB-55 (2)**.

^{1}H-NMR (600 MHz; in CD_3OD) at 10 °C, signal assignment from ^{1}H,^{13}C-HSQC, ^{1}H,^{13}C-HMBC, ^{1}H,^{1}H-COSY and ^{1}H,^{1}H-ROESY: δ [ppm] = 1.11 (t, *J*=7.5 Hz, $H_3C\text{-}3^2$), 1.17 (s, $H_3C\text{-}7^1$), 1.99 (s, $H_3C\text{-}13^1$), 2.01 (s, $H_3C\text{-}17^1$), 2.34 (s, $H_3C\text{-}2^1$), 2.45 (t, *J*=6.8 Hz, $H_2C\text{-}12^2$), 2.52 (m, $H_2C\text{-}3^1$); 2.57 (m, H_AC-15), 2.75 (m, $H_AC\text{-}8^2$), 2.77 (m, $H_2C\text{-}12^1$), 2.94 (d, *J*=14.6 Hz, H_BC-15), 2.95 (d, *J*=15 Hz, H_AC-5), 3.15 (d, *J*=15.2 Hz, H_BC-5), 3.21 (dd, *J*=6.0/18.2 Hz, $H_BC\text{-}8^2$), 4.11 (dd, *J*=4.9/8.9 Hz,

HC-16), 4.63 (dd, *J*=2.0/6.8 Hz, HC-10), 5.37 (dd, *J*=2.3/11.7 Hz, H_AC-18^2), 6.15 (dd, *J*=2.3/17.8 Hz, H_BC-18^2), 6.49 (dd, *J*=11.6/17.6 Hz, HC-18^1), 9.49 (s, HC-20).
^{13}C-NMR (150 MHz; in CD_3OD) at 10 °C, signal assignment from ^{1}H,^{13}C-HSQC and ^{1}H,^{13}C-HMBC: δ [ppm] = 9.3 (2^1), 10.0 (13^1), 13.0 (17^1), 16.6 (3^2), 18.2 (3^1), 20.3 (7^1), 22.3 (12^1), 31.0 (15), 32.8 (5), 34.6 (10), 38.7 (12^2), 48.1 (8^2), 50.4 (7), 62.7 (16), 116.4 (13), 119.6 (18^2), 121.0 (12), 123.5 (9), 125.3 (14), 127.6 (18^1), 128.4 (8), 128.7 (3), 129.5 (18), 131.2 (1), 133.8 (2), 136.4 (4), 157.6 (17), 175.8 (19), 178.3 (20), 180.9 (12^3), 183.8 (9), 188.8 (6), 200.4 (8^1).

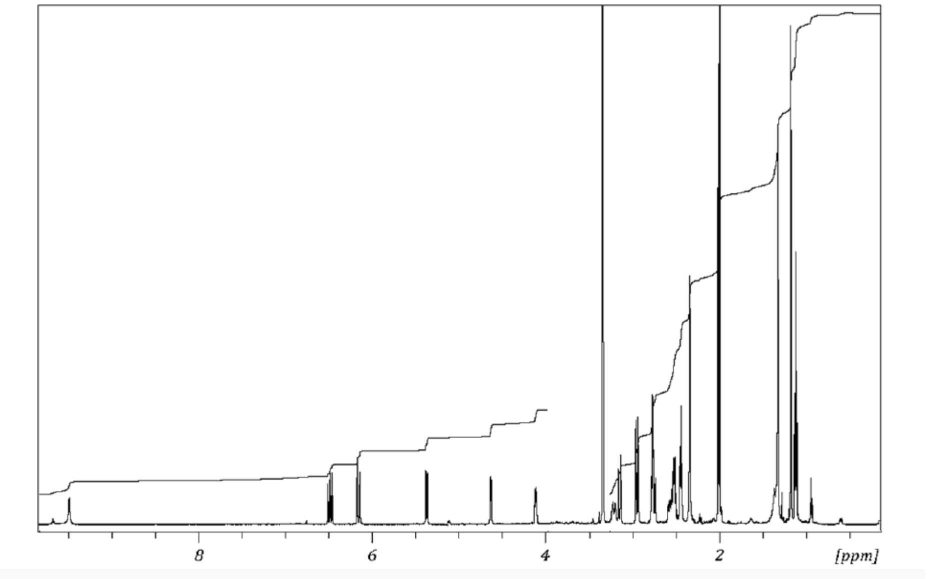

Figure S6. 600 MHz ^{1}H NMR spectrum of ***Pa-i*PB-55 (2)** (CD_3OD, 10 °C).

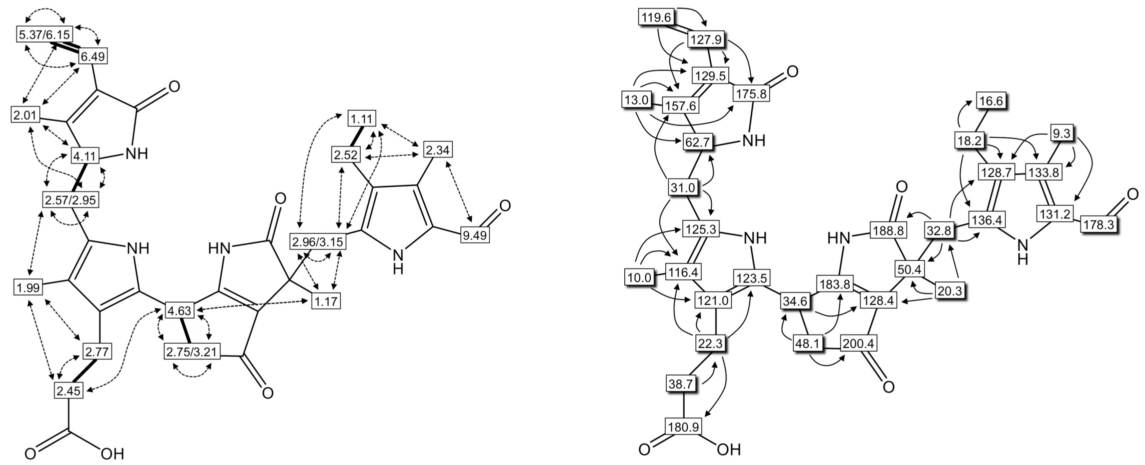

Figure S7. NMR chemical shift data of *Pa-i*PB-55 depicted in a graphical representation of the derived molecular structure of **2** (600 MHz-NMR, CD_3OD, 10 °C). Left: Homonuclear correlations from ^{1}H,^{1}H-ROESY experiment (dashed lines); bold bonds represent spin systems derived from the ^{1}H,^{1}H-COSY spectrum. Right: Heteronuclear correlations based on assignments and obtained from ^{1}H,^{13}C-HSQC spectra (shaded boxes) and ^{1}H,^{13}C-HMBC experiments (arrows).

Transformation of *Pa-i*PB-45 (1) into iminoester 3.

Transformation: 2 µL triethylamine were added to 2 mg *Pa-i*PB-45 (1) in 4 mL methanol at 0 °C under argon. The solution was stirred for 30 min at 0 °C. 2.5 mg Benzotriazol-1-yloxy-tris(dimethylamino)-phosphonium hexafluorophosphate (BOP) were added and then the mixture was stirred for 17 h at RT under argon atmosphere and under light protection. The resulting solution was evaporated under reduced pressure.

*Isolation of Pa-iPB-45 iminoester (**3**)*: The residue was re-dissolved in 3 mL methanol/H_2O (1:2, v/v) and isolated by semipreparative HPLC: fow rate: 0.5 mL*min^{-1}; solvent A: 50 mM potassium phosphate buffer (pH 7), solvent B: methanol; solvent composition A/B (v/v): 0-5 min: 45/55; 5-20 min: 45/55 to 42/58; 20-23 min: 42/58 to 0/100; 23-23 min: 0/100; 26-30 min: 0/100 to 45/55. 15 consecutive HPLC runs were performed and fractions from R_t = 16 to 18 min containing *Pa-i*PB-45 iminoester were collected. The combined fractions were diluted with 80 mL H_2O and applied to a preconditioned SepPak-cartridge (5g). After washing with 20 mL H_2O the *Pa-i*PB-45 iminoester was eluted with 6 mL methanol. After the removal of the solvents in vacuo, 443 µg pure *Pa-i*PB-45 iminoester (21.6% yield) was obtained.

Spectroanalytical data of *Pa-i*PB-45 iminoester (3).

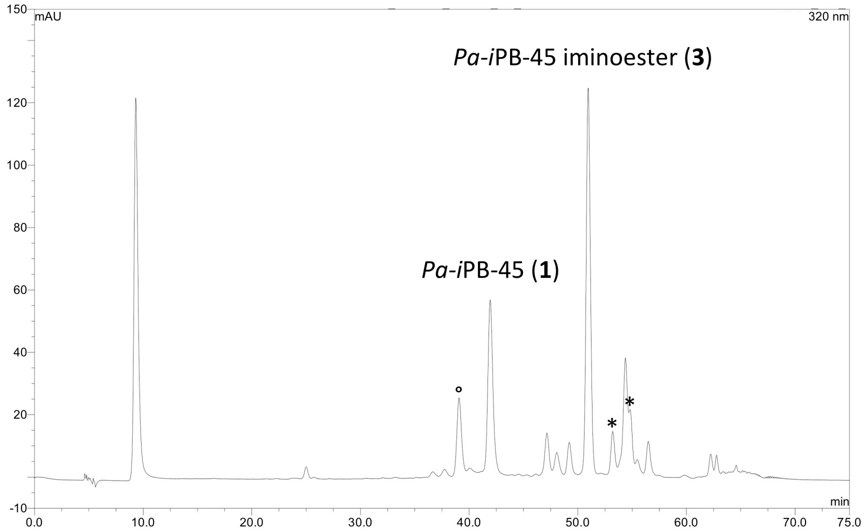

Figure S8. HPLC-analysis of the crude reaction mixture after 17 h of treatment of *Pa-i*PB-45 (1) with BOP (detection at 320 nm), *Pa-i*PB-45 and the *Pa-i*PB-45 iminoester (3) are highlighted, (°) is an unidentified isomeric form of *Pa-i*PB-45, (*) are methylesters of *Pa-i*PB-45 based on LC/MS analysis.

ESI-MS *m/z* (%): 693.2 (4, $[M\text{-}H+2K]^+$); 677.2 (10, $[M\text{-}H+K+Na]^+$); 661.3 (11, $[M\text{-}H+2Na]^+$); 655.3 (81, $[M+K]^+$); 641.3 (8), 640.3 (38), 639.3 (100, $[M+Na]^+$); 619.3 (4), 618.3 (18), 617.3 (48, $C_{34}H_{41}N_4O_7^+$, $[M+H]^+$); 599.3 (5, $[M\text{-}H_2O+H]^+$); 589.3 (4, $[M\text{-}CO+H]^+$); 585.3 (2, $[M\text{-}CH_4O+H]^+$); 494.2 (35, $[M\text{-}C_7H_9NO+H]^+$); 476.2 (8, $[M\text{-}C_7H_9NO\text{-}H_2O+H]^+$); 466.2 (6, $[M\text{-}C_7H_9NO\text{-}CO+H]^+$); 462.2 (3, $[M\text{-}CH_4O\text{-}C_7H_9NO+H]^+$).

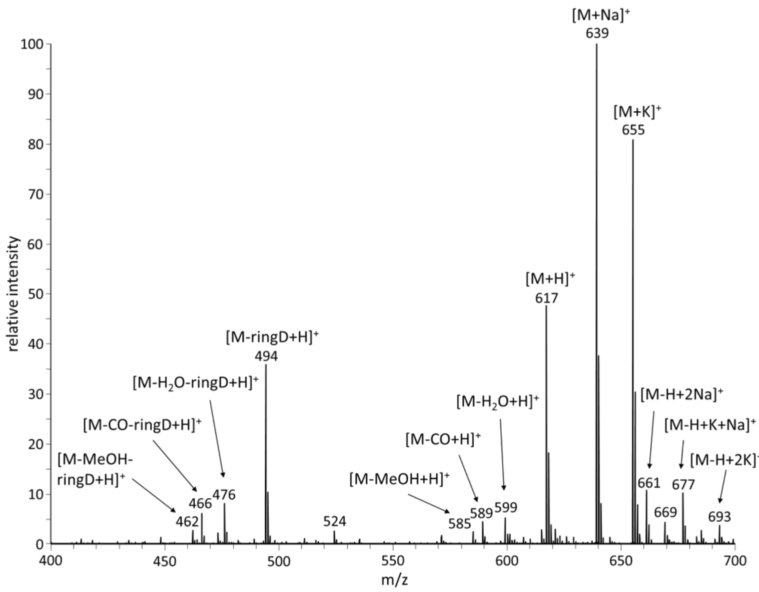

Figure S9. ESI mass spectrum of ***Pa-i*****PB-45 iminoester (3)**.

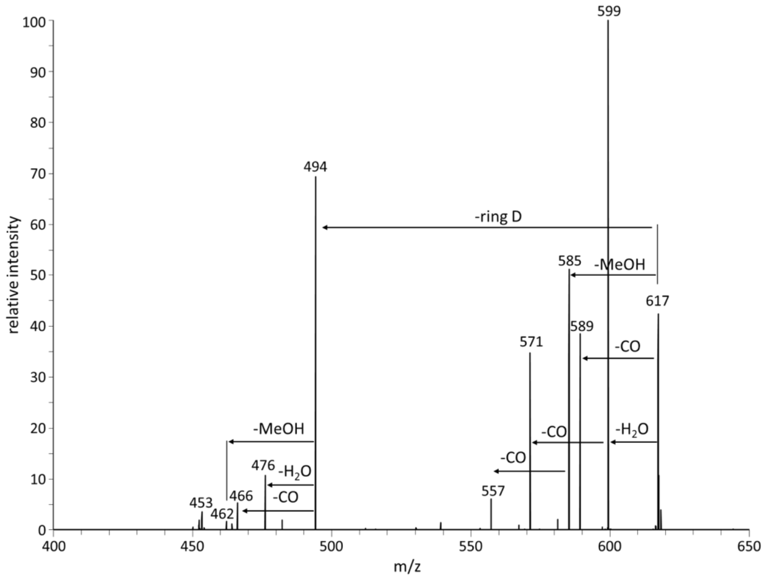

Figure S10. Fragmentation the isolated $[M+H]^+$ ion of the ***Pa-i*****PB-45 iminoester (3)**.

^{1}H-NMR (600 MHz; in CD_3OD) at 5 °C, signal assignment from ^{1}H,^{13}C-HSQC, ^{1}H,^{13}C-HMBC, ^{1}H-^{1}H-COSY and ^{1}H-^{1}H-ROESY: δ [ppm] = 1.55 (s, H_3C-7^1), 1.87 (s, H_3C-13^1), 1.98 (s, H_3C-17^1), 2.28 (s, H_3C-2^1), 2.43 (m, H_AC-12^2), 2.54 (m, H_AC-3^1), 2.56 (m, H_AC-15), 2.63 (m, H_BC-3^1), 2.84 (m, H_2C-12^1), 2.87 (m, H_AC-8^2), 2.92 (m, H_BC-15), 2.96 (m, H_BC-8^2), 3.05 (m, H_AC-5), 3.17 (m, H_BC-12^2), 3.20 (m, H_BC-5), 3.53 (t, J = 7.3 Hz, H_2C-3^2), 3.67 (s, H_3C-6^2), 4.09 (dd, J = 5.2/8.3 Hz, HC-16), 4.98 (HC-10, superimposed by 5.04 (H_2O)), 5.35 (dd, J = 2.3/11.6 Hz, H_AC-18^2), 6.10 (dd, J = 2.3/17.8 Hz, H_BC-18^2), 6.45 (dd, J = 11.6/17.8 Hz, HC-18^1), 9.42 (s, HC-20).

^{13}C-NMR (150 MHz; in CD_3OD) at 5 °C, signal assignment from ^{1}H,^{13}C-HSQC and ^{1}H,^{13}C-HMBC: δ [ppm] = 8.9 (2^1), 8.9 (13^1), 12.5 (17^1), 22.0 (7^1), 22.4 (12^1), 28.1 (3^1), 30.2 (15), 32.4 (5), 35.9 (12^2), 38.4 (8^2), 39.6 (10), 48.1 (7), 52.3 (6^2), 61.7 (16), 62.5 (3^2), 116.6 (13), 118.8 (18^2), 119.2 (12), 122.6 (3), 122.6 (11), 123.5 (14), 124.8 (8), 126.8 (18^1), 128.7 (18), 130.4 (1),

133.9 (2), 137.2 (4), 157.0 (17), 169.0 (9), 175.0 (19), 176.8 (12^3), 177.6 (6), 177.6 (20), 205.2 (8^1).

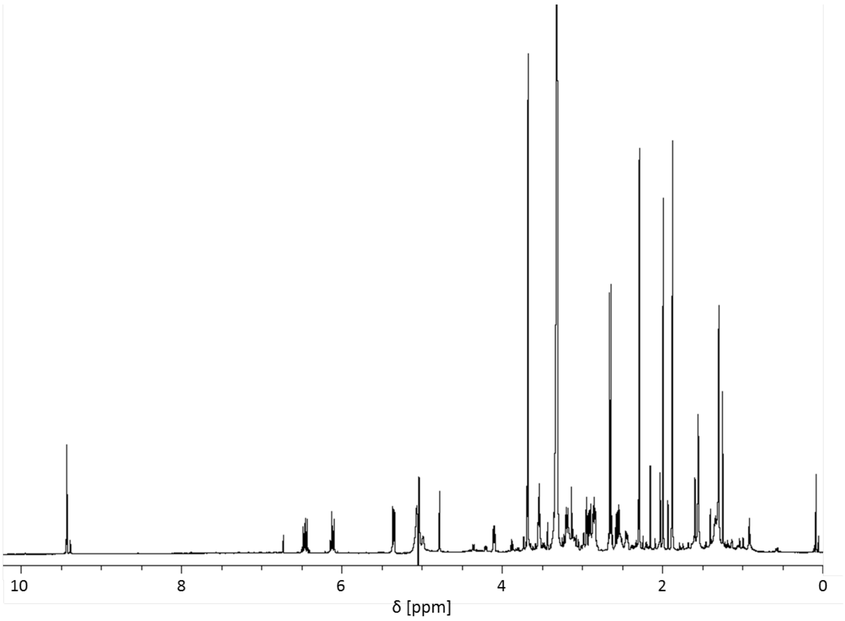

Figure S11. 600 MHz ^{1}H-NMR spectrum of *Pa-i*PB-45 iminoester (CD_3OD, 5 °C)

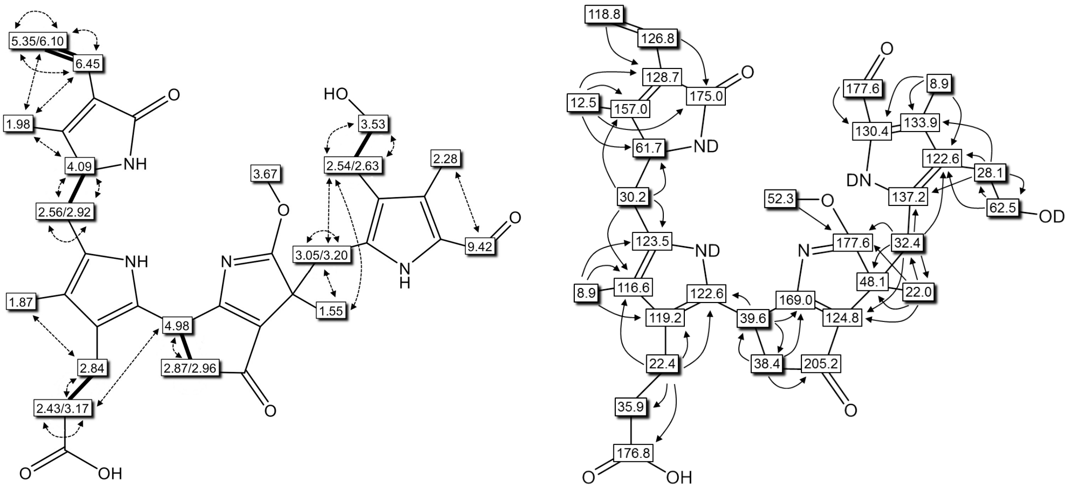

Figure S12. NMR chemical shift data of *Pa-i*PB-45 iminoester depicted in a graphical representation of the derived molecular structure of **3** (600 MHz-NMR, CD_3OD, 5 °C). Left: Homonuclear correlations from ^{1}H,^{1}H-ROESY experiment (dashed lines); bold bonds represent spin systems derived from the ^{1}H,^{1}H-COSY spectrum. Right: Heteronuclear correlations based on assignments and obtained from ^{1}H,^{13}C-HSQC spectra (shaded boxes) and ^{1}H,^{13}C-HMBC experiments (arrows).

References.

1. Gottlieb, H. E., Kotlyar, V. & Nudelman, A. (1997) NMR chemical shifts of common laboratory solvents as trace impurities, *J. Org. Chem.* **62**, 7512-7515.
